# Hyperoxia Leads to Transient Endocrine Alterations in the Neonatal Rat During Postnatal Development

**DOI:** 10.3389/fped.2021.723928

**Published:** 2021-10-26

**Authors:** Mirjam Kowallick, Meray Serdar, Boyka Markova, Eva Salveridou, Ursula Felderhoff-Müser, Dagmar Führer-Sakel, Heike Heuer, Ivo Bendix, Monia Vanessa Dewan

**Affiliations:** ^1^Department of Paediatrics I—Neonatology and Experimental Perinatal Neurosciences, University Hospital Essen, University of Duisburg-Essen, Essen, Germany; ^2^Department of Endocrinology, Diabetes and Metabolism, University Hospital Essen, University of Duisburg-Essen, Essen, Germany

**Keywords:** preterm infants, pituitary gland, hormones, neurodevelopment, hyperoxia

## Abstract

**Introduction:** High oxygen concentrations have been identified as one factor contributing to the pathogenesis of the retinopathia of prematurity, chronic lung disease of the preterm infant and preterm brain injury. Preterm infants also show short- and long-term alterations of the endocrine system. If hyperoxia is one pathogenetic factor has not been investigated yet. With regard to the high prevalence of neurodevelopmental impairments in preterm infants, the hypothalamus-pituitary-thyroid (HPT) axis, the hypothalamus-pituitary-adrenal (HPA) axis and the hypothalamus-pituitary-somatotropic (HPS) axis are of special interest due to their important role in neurodevelopment.

**Objective:** The aim of this study was to investigate the effect of hyperoxia on the endocrine system in the neonatal rat by analyzing the activities of the HPT, HPA and HPS axes, respectively.

**Methods:** Three-days old Wistar rats were exposed to hyperoxia (oxygen 80%, 48 h). On postnatal day 5 (P5) and P11, transcript levels of thyroid-stimulating hormone (TSH), proopiomelanocortin and growth hormone (GH) were analyzed in pituitary sections by *in situ* hybridization. Serologic quantification of TSH and thyroxine (T4), adrenocorticotropic hormone and GH were performed by Multiplex analysis and Enzyme-linked Immunosorbent Assay.

**Results:** At P5, significantly lower GH levels were observed in pituitaries (mRNA) and in sera of rats exposed to hyperoxia. Serum TSH was significantly elevated without changes in T4.

**Conclusion:** This is the first study demonstrating transient endocrine alterations following hyperoxia in the neonatal rat making oxygen a possible contributor to the pathogenesis of endocrine alterations seen in preterm infants. Considering the detrimental multi-organ effects of hyperoxia on the immature organism, a rational use of therapeutic oxygen in the treatrnent of preterm infants is of utmost importance.

## Introduction

Fetal development occurs under relative hypoxic conditions (PaO_2_ of ~25 mm Hg) *in utero*. Consequently, the transition to the relative hyperoxic extra-uterine environment (PaO_2_ of 70 mm Hg) exposes a preterm born infant to oxidative stress in a period when the antioxidant system is still immature (1, 2) with detrimental effects on the developing child. At the same time, supplemental oxygen is the most used therapeutic agent in the care of preterm born infants in the treatment and prevention of hypoxia (3). Thus, understanding its effects on the immature infant is indispensable.

It is well-established that high levels of oxygen contribute to the development of the retinopathia of prematurity and chronic lung disease of the preterm infant (4, 5). Furthermore, clinical and pre-clinical studies showed, that hyperoxia is one factor contributing to the development of preterm brain injury (2, 6). In a rat model of hyperoxia-induced brain injury, our study group could repeatedly show that the exposition of rat pups to 2-48 h of hyperoxia (FiO2 80%) leads to transient hypomyelination and long-term cognitive deficits by cellular degeneration, oxidative stress and inflammation (7–13).

Preterm infants show short- and long-term endocrine alterations. To which extent hyperoxia effects the endocrine system of the immature organism, has not been investigated yet. Considering the increased risk of neurodevelopmental impairment of preterm infants (14, 15) and the effect of hyperoxia on neurodevelopment, alterations in the hypothalamus-pituitary-thyroid (HPT) axis, the hypothalamus-pituitary adrenal (HPA) axis and the hypothalamus-pituitary-somatotropic (HPS) axis are of special interest due to the role of thyroid hormone, glucorticoids and growth hormone/Insulin-like growth factor in central nervous system development and function (16–18).

As part of the HPT axis, thyroid-stimulating hormone (TSH) regulates the production of the thyroid hormones the prohormone thyroxine (T4) and its active form triiodothyronine (T3), which are essential factors in neuronal migration, proliferation and differentiation, myelination and synaptogenesis (19). Preterm infants born before 32 weeks' gestation might develop transient hypothyroxinemia of prematurity (THOP) which is characterized by low T4 levels and normal TSH. THOP normally resolves within the first 3 weeks of life (20, 21). A small number of infants were shown to exhibit a transient TSH elevation due to a delayed thyroid-releasing hormone (TRH) surge, which physiologically occurs at the transition from intra- to extrauterine life in term born infants (21). Besides developmental immaturity, factors like illness and drugs might play a role in the pathogenesis of THOP and the delay of the TRH surge (20). Prematurity might have an impact on long-term thyroid function as a recent study by Posod et al. showed significantly higher TSH levels in very preterm born children at preschool age compared to term born children (22). The effects of thyroid dysfunction in preterm infants on long-term neurodevelopment remains controversial; while researchers of some studies did not find any effects (23), others found inferior neurodevelopmental outcome associated with lower neonatal thyroid hormone levels (24, 25). In a study by Ng et al., plasma free T4 levels in the lowest quartile in a cohort of very preterm infants were associated with lower fractional anisotropy in diffusion tensor imaging as sign of poorly organized microstructure at term equivalent age (26). Nevertheless, prophylactic thyroid hormone replacement in extremely preterm infants has not been proven to exert any beneficial effects (27, 28).

Adrenocorticotropic hormone (ACTH) and its precursor protein proopiomelanocortin (POMC) are part of the HPA axis which is important for fetal development and transition at birth (29). In very preterm infants, the HPA axis is still immature which is reflected by an impaired stress reaction (20, 30). Long-term alterations of the HPA axis with increased glucocorticoid bioactivity and its negative effects on growth, metabolism, body composition, and neurodevelopment have been described (31). Studies showed that ACTH promotes the differentiation of oligodendroglial progenitor cells to oligodendrocytes accompanied by increased myelination and protects the progenitor cells from excitotoxic and inflammation-related damage (32). In a rat model of intraventricular hemorrhage, ACTH showed neuroprotective effects in newborn rats (33).

Growth hormone (GH) can be detected in the human fetus by 9 weeks' gestation and levels rise until term (34). With GH receptors being present in the fetal and juvenile brain and especially enriched in cortical, hypothalamic and hippocampal neurons, GH is assumed to influence growth and development of the CNS, but exact mechanisms are still unknown (18). Preterm birth seems to disrupt the normal GH release pattern as very preterm infants show an increased GH secretory activity (35). Following a study by Scratch et al., higher GH levels in the first 6 weeks of life in a cohort of very preterm infants were associated with cognitive deficits at the age of 7 years (36).

We hypothesized that hyperoxia leads to alterations in the activity of the HPT, HPA and HPS axes, respectively. Rat pups were exposed to 48 h hyperoxia from postnatal day 3 (P3) to P5, a period when the rat's endocrine system including the pituitary is still immature as anatomical and functional maturation occurs during the first 2 weeks of life (37). We chose that period based on our studies on hyperoxia-induced brain injury where we focused on white matter disturbances at a time when the maturation state of oligodendrocytes is comparable to that of extremely preterm infants (GA < 28 weeks of gestation) (38). We then analyzed the hormones of interest in pituitaries (TSH, POMC, GH) by mRNA *in situ* hybridization (ISH) and in sera (TSH and T4, ACTH, GH) by using a luminex-multiplex assay and Enzyme-linked Immunosorbent Assay (ELISA) at P5 and P11.

## Materials and Methods

### Animals and Experimental Design

All animal procedures were approved by the local animal welfare committee by the State Agency for Nature, Environment and Consumer Protection North Rhine-Westphalia and performed according to the guidelines of the University Hospital Essen.

Three-days old (P3) Wistar rat pups were placed in an oxygen chamber (OxyCycler, Bio-Spherix, Lacona, NY, USA) with an oxygen level of 80% for 48 h. The control group was kept under normoxic conditions (21% oxygen). Both groups were accompanied by their lactating dams. After a period of 24 h, dams were exchanged in order to avoid prolonged exposition to hyperoxia. Animals were kept under controlled light cycle (12 h light, 12 h dark). In total, 34 pups (P5: 9 NO group, 8 HO group; P11: 8 NO group, 9 HO group) were enrolled derived from four litters. To ensure heterogeneity, pups were derived from two litters per experiment and randomly assigned to the different treatment groups. All groups were sex- and weight matched. Increase of bodyweight was raised regularly and showed a comparable pattern between the study groups.

Pups were sacrificed on P5 (17 pups, 2 litters, 8 female, and 9 male) and P11 (17 pups, 2 litters, 9 female, and 8 male) by decapitation. The rationale for this litter size was based on our experience with studies on hyperoxia-induced brain injury, where these litter sizes are necessary to see significant effects as the exposition to 48 h hyperoxia only leads to subtle and diffuse changes. At P5 mean weight was 10.41 g (SD 0.457) for the NO group and 10.85 g for the HO group (SD 0.566), at P11 mean weight in the NO group was 24.78 g (SD 1.595) and 23.33 g in the HO group (SD 1.668). There were no significant weight differences at P5 (*p* = 0.1) and at P11 (*p* = 0.09).

Brains were removed and pituitaries were embedded in Tissue-Tek medium (Sakura Finetek, Torrance, CA, USA) and frozen on dry ice. Pituitary sections (14 μm) were cut on a cryostat (Leica, Bentheim, Germany), mounted on supefrost plus slides and stored at −80 until further processing.

**Figure 1:**
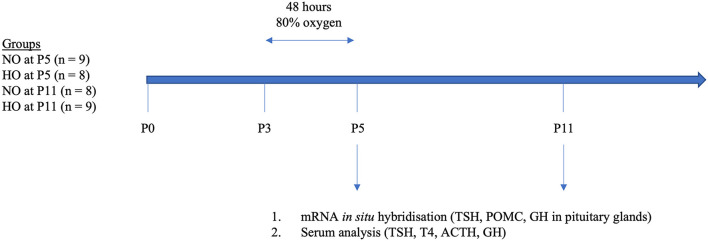
Schematic presentation of the study design. At postnatal day 3 (P3) rat pups were exposed to 48 h hyperoxia (HO, 80% oxygen). The control group was kept under normoxic conditions (room air). At P5 and P11, rat pups were sacrificed and pituitaries were removed and blood samples were conserved. We then analyzed TSH, POMC, and GH in pituitaries by mRNA *in situ* hybridization and TSH, T4, ACTH, and GH in sera by multiplex analysis (ACTH, TSH, T4) and ELISA (GH).

Trunk blood was collected from the site where the animal was decapitated. Serum was removed after centrifugation for 10 min at 1,000 × g. Samples were stored in polypropylene tubes at −80°C. For a schematic outline of the study protocol please see Figure 1.

### *In situ* Hybridization Analysis of Pituitary TSH, POMC, and GH Expression

*ISH* histochemistry was carried out as described before (39).

In brief, frozen 14 μm pituitary sections were air-dried, fixed in 4% phosphate-buffered PFA solution (pH 7.4) for 1 h at RT, rinsed with PBS, permeabilized with 0.4% phosphate-buffered Triton X-100 for 10 min, and then washed with PBS. Acetylation was carried out in 0.1 M triethanolamine (pH 8.0) containing 0.25% (v/v) acetic anhydride. After 10 min, sections were rinsed with PBS, dehydrated with 50 and 70% ethanol, and air-dried. CDNA fragments corresponding to nt 190-443 of rat TSH (accsession no. M10902.1), nt 56-526 of rat POMC (accession no. AH002232) and nt 248-445 of rat GH (accession no. U62779.1) were used as templates for the synthesis of digoxigenin-labeled riboprobes by *in vitro* transcription. After synthesis and purification, digoxigenin-labeled riboprobes were diluted in hybridization buffer containing 50% formamide, 10% dextran sulfate, 0.6 M NaCl, 10 mM Tris/HCl (pH 7.4), 1 × Denhardt's solution, 100 μg/ml sonicated salmon sperm DNA, 1 mM EDTA, and 10 mM dithiotreitol to a final concentration 5 ng/μl cRNA for all probes.

After applying the hybridization mix, sections were coverslipped and incubated at 52°C overnight in a humid chamber. After hybridization, sections were rinsed in 2 × standard saline citrate (0.3 M NaCl and 0.03 M sodium citrate, pH 7.0) and subsequently treated with ribonuclease A/T1 at 37°C for 30 min. Additional washing steps for 20 min were carried out in 1 ×, 0.5 ×, 0.2 × standard saline citrate at RT followed by incubation in 0.2 × standard saline citrate at 65°C for 1 h. Sections were rinsed in B1 (100 mM Tris-HCl pH 7.5, 150 mM NaCl, pH 7.5), blocked for 90 min in buffer B1 containing 10% milk-powder and then incubated overnight at 4°C with anti-digoxigenin antibody conjugated with alkaline phosphatase (1:500 dilution; Roche) in B1 were washed with B1 and B3 (100 mM Tris-HCl pH 9.5, 100 mM NaCl, 50 mM MgCl2). Staining proceeded in substrate solution containing nitroblue tetrazolium chloride (75 mg/ml; Sigma), X-Phosphate (5-bromo-4-chloro-3-indolyl phosphate, 50 mg/ml, Sigma), 100 mM Tris, 100 mM NaCl and 50 mM MgCl_2_ for 4.5 h (TSH), 3.5 h (POMC) and 2 h (GH). Experiments were carried out using the respective sense probes that did not produce any ISH signals.

Image analysis was carried out with ImageJ (National Institutes of Health, Java 1.8.0). Images were converted to 8-bit and inverted using the “invert” tool in ImageJ. The total area of the anterior pituitary, defined as total field of view area, was outlined for each image with the tool “polygon,” excluding artifacts. Studies showed an equal distribution of somatotropes and corticotropes in the anterior pituitary in coronal sections (40, 41), while to the best knowledge of the authors no publication to the distribution of thyrotropes exists. For quantification, the total area was measured in square pixels. To differentiate background staining, the intensity of the background signal for each hormone was quantified. After defining the background signal, this was excluded by using the plugin “threshold.” The remaining area, called “threshold area,” represents the positive mRNA signals for each hormone. To quantify mRNA expression, the “threshold area” was measured in square pixels, and the percentage of “threshold area” of the total area was calculated.

The number of analyzed images ranged from 6-8 per group at P5 and 6-9 at P11 due to insufficient staining quality or to extended artifacts.

### Serological Examination of TSH and T4, ACTH, and GH

For simultaneous quantification of TSH and ACTH serum concentrations, the MILLIPLEX^®^ MAP Rat pituitary magnetic bead panel (EMD Millipore Corporation, Billerica, USA.; # RPTMAG-86K) was used. Serum samples were diluted 1:3 in the diluted Serum Matrix provided in the kit. GH was determined in sera using a commercial Rat/Mouse ELISA kit according to the manufacturer's instructions (EMD Millipore Corporation, Billerica, USA.; # EZRMGH-45K). Serum samples were diluted 1:2 in the Assay Buffer provided. Serum T4 was measured with the MILLIPLEX^®^ MAP Rat thyroid hormone magnetic bead panel (EMD Millipore Corporation, Billerica, USA.; # RTHYMAG-30K) using a 1:6 dilution. One probe of the NO group at P5 was not measured for T4 due to insufficient probe volume. All kits were used according to the manufacturer's instructions. Quality control samples provided by the kits were measured within the expected parameters. Standard curves ranged from 3.2–10,000 pg/ml for the ACTH and TSH assay (accuracy 88, 90%), 0.07-50 ng/ml for the GH assay (accuracy 96-101%) and 823-200,000 pg/ml for the T4 assay (accuracy 131%). All samples except of 1 in the ACTH assay (P11, HO group, excluded for statistical analysis) were in the range of the standard curve.

### Statistical Analysis

Statistical analysis was performed with Prism 6 (GraphPad Software, San Diego, CA, USA). Graphical data are presented as mean ± standard deviation. Normality distribution was confirmed with the D'Agostino-Pearson test. Unpaired student's *t*-test was applied to determine differences in positive area in ISH experiments and in serum concentrations of the hormones. *p-*values ≤ 0.05 were considered as statistically significant.

## Results

### Decreased Pituitary GH Transcript Levels Following Hyperoxia

**Figure 2:**
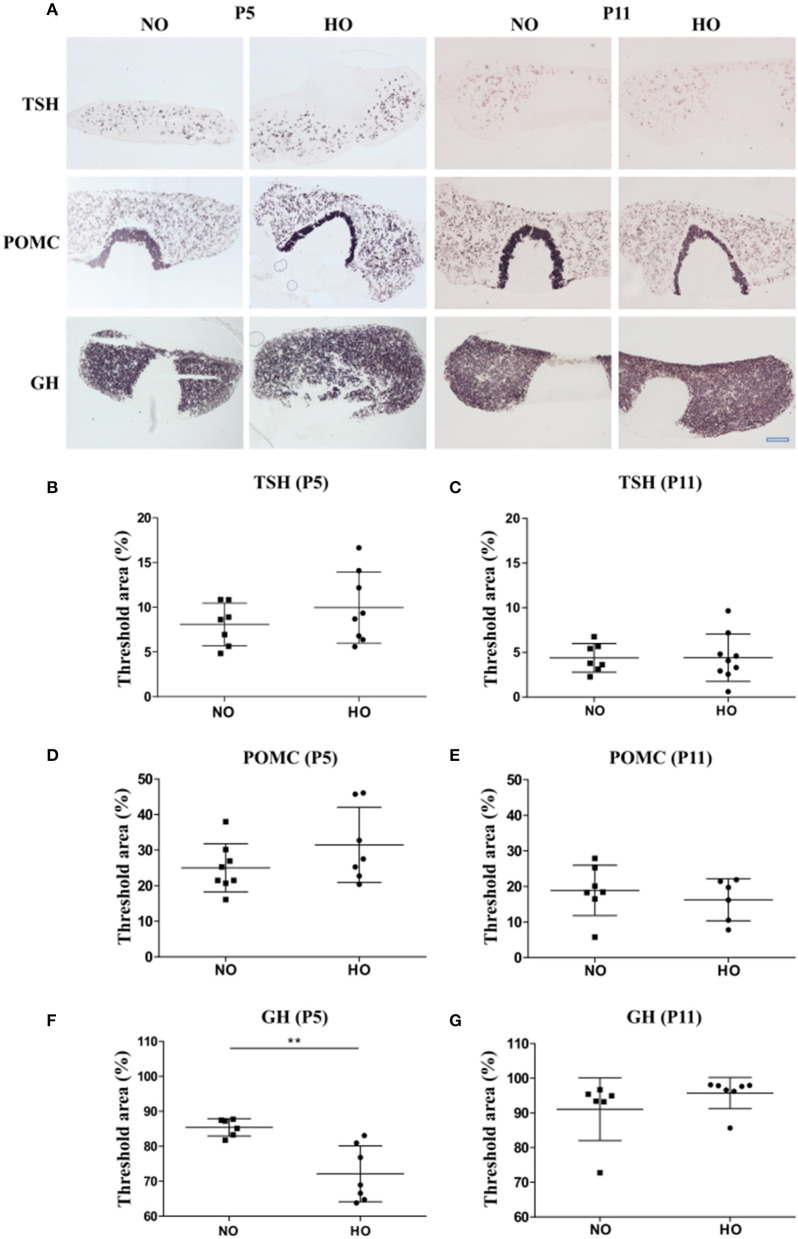
**(A)** mRNA expression of different pituitary hormones of 5- and 11-days (P5 and P11) old Wistar rat pups after 48 h of hyperoxia (HO, 80% oxygen) and in the control group (NO, 21% oxygen). 14 μm thick sections of pituitaries were hybridized with digoxigenin-labeled cRNA probes for TSH, POMC and GH. Scale bar = 500 μm. **(B-E)** Hybridization signal intensities were quantified and expressed as percentage of threshold area (%) of the total area (anterior pituitary). The threshold was set for each hormone to differentiate specific hybridization signals from background signals. No differences were found for TSH and POMC between NO- and HO-groups at P5 or P11 **(F)**. A significant lower threshold area was observed for GH in the HO group compared to the control group at P5 without differences at P11 **(G)**. Mean ± SD. **p < 0.01. *n* = 6-9 per group.

In this study, 3-days old Wistar rat pups were exposed to 48 h hyperoxia (HO, oxygen 80% from P3-P5), while the control group was under normoxic conditions (NO, oxygen 21%). To investigate the effect of hyperoxia on the activity of the HPT, HPA and HPS axes, mRNA expression of TSH, POMC and GH was analyzed in pituitary sections of 5- and 11-days old Wistar rat pups by ISH using digoxigenin-labeled cRNA probes for TSH, POMC and GH (Figure 2A). Quantification of ISH signals in the anterior pituitary showed significant lower GH mRNA levels in the HO group compared to the NO group at P5 (Figure 2F), while no differences were found at P11 (Figure 2G). ISH with mRNA probes specific for TSH and POMC in pituitaries did not show significant differences in signal intensities at P5 or P11 (Figures 2B-E).

### Transient Alterations of GH and TSH Serum Levels Following Hyperoxia

**Figure 3:**
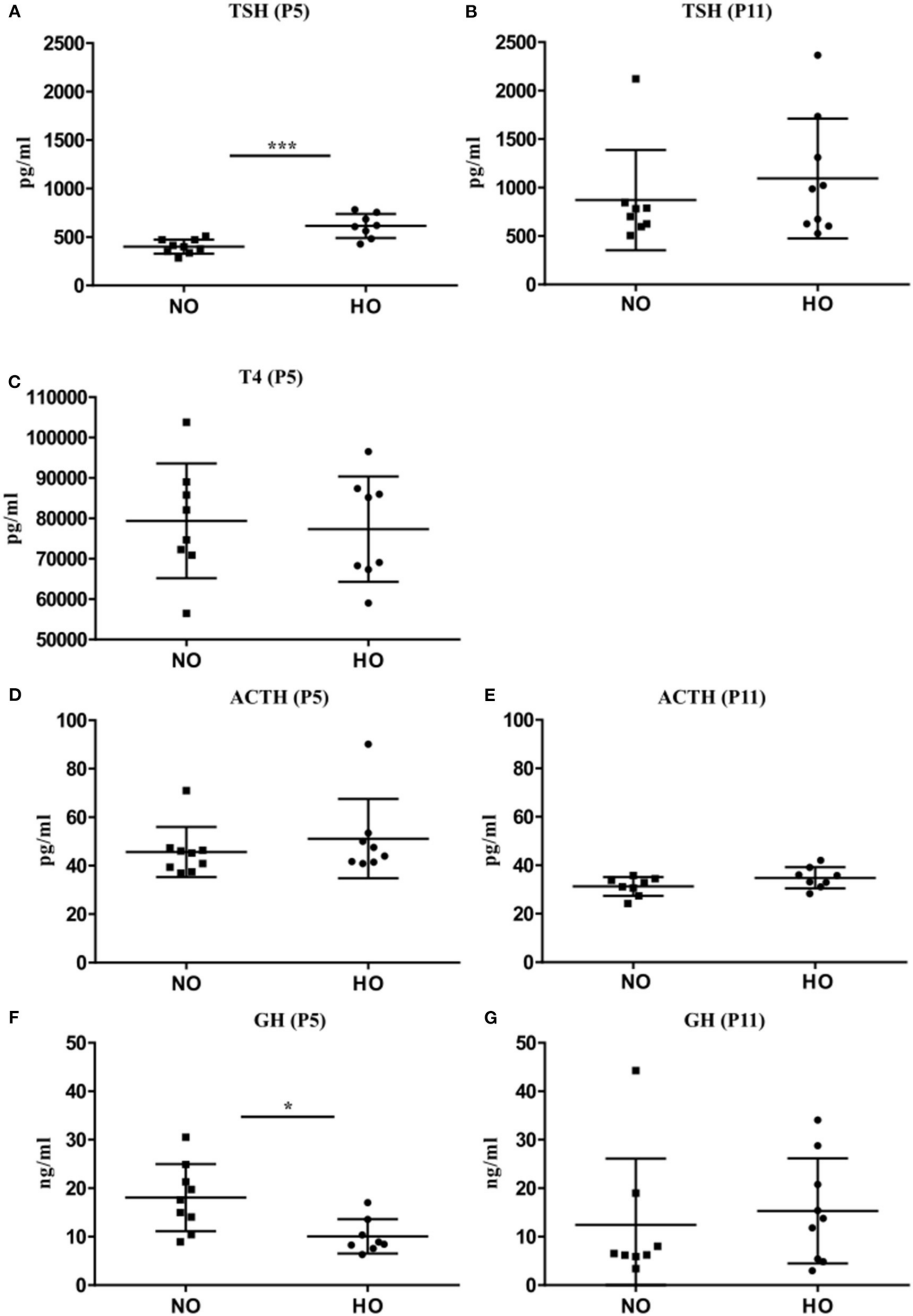
Serum concentration of TSH, ACTH, and GH were determined at P5 and P11 after 48 h hyperoxia (80% oxygen from P3-P5). **(A)** TSH levels at P5 were significantly higher in the HO group compared to the NO group but no difference could be noted at P11 **(B)**. **(C)** Analysis of serum T4 levels at P5 also did not reveal any changes. No differences in POMC levels at P5 **(D)** or P11 **(E)** were found. **(F)** Significant lower GH levels in the HO group were detected at P5, but not at P11 **(G)**. Mean ± SD. ^*^p < 0.05, ^***^p < 0.001, *n* = 7-8 rats/group.

To confirm the analyses of mRNA expression in pituitaries, corresponding analyses of TSH, ACTH and GH serum levels were performed in the same rat pups on P5 and P11 by a bead-based fluorescence MILLIPLEX® assay/Luminex (TSH and ACTH) and ELISA (GH). Significantly higher serum TSH levels could be detected in rats after hyperoxia (Figure 3A) only at P5 but not at P11 (Figure 3B). In order to assess whether the early rise in TSH serum levels affect thyroid hormone production and secretion, we also measured T4 levels at P5 by a bead-based fluorescence MILLIPLEX® assay/Luminex assay. However, no significant differences could be detected (Figure 3C). Serum ACTH levels were not different between NO and HO groups at P5 and P11 (Figures 3D,E). In correspondence to the decreased GH transcript levels, serum GH was significantly lower in the HO group at P5 without differences at P11 (Figures 3F,G).

## Discussion

Although oxygen is an indispensable therapeutic agent in neonatal care, its use is linked to the development of several prematurity associated morbidities such as retinopathia, chronic lung disease and brain injury (2, 4–6). If hyperoxia also effects the immature endocrine system, has not been investigated yet.

Based on our studies on hyperoxia-induced brain injury in the neonatal rat, which is characterized by subacute myelination deficits and long-term cognitive impairments (7–13), we focused on the hypothalamus-pituitary-thyroid (HPT) axis, the hypothalamus-pituitary adrenal (HPA) axis and the hypothalamus-pituitary-somatotropic axis (HPS) due to their crucial role in neurodevelopment (16–18).

We studied the expression of the pituitary hormones TSH, POMC/ACTH and GH, as changes in the production and secretion of these hormones would provide a first hint regarding to hyperoxia-induced alterations of the activities of the HPT, HPA and HPS axes, respectively. We found transient alterations of serum TSH and of pituitary GH mRNA as well as GH serum levels at P5 that confirm our hypothesis.

TSH is a glycoprotein, that consists of the two subunits TSHα and TSHß. While the subunit TSHα is common with the pituitary gonadotropins, TSHß, which was detected in our mRNA ISH, is specific for thyrotropes and necessary for the bioactivity of TSH. The transcription of the ß-subunit and secretion of TSH is mainly modulated by thyroid hormones via a negative feedback mechanism and by the thyrotropin releasing hormone (TRH) *via* a positive feed-forward mechanism (42). Divergent results for TSH mRNA levels in pituitaries and protein concentrations in sera might be explained by a short or ultra-short negative feedback loop based on TSH autoregulation at the level of the pituitary (43).

Elevated TSH levels in sera might be explained by the modulating effects of TRH neurons which are localized in the paraventricular nucleus of the hypothalamus. These neurons receive afferents from various brain regions. Besides the thyroid hormone mediated negative feedback mechanism, TRH neurons are activated by different internal (e.g., energy status) and environmental (e.g., light, cold) stimuli (44). The effect of hyperoxia on TRH transcription and release remains to be determined. Increased TSH levels might also be explained by a transient depressed thyroid function. Although T4 levels were not altered, there is still the possibility of subclinical hypothyroidism (45). Furthermore, altered T3 levels cannot be excluded, because physiologically low T3 levels at P5 prevented a valid quantification in this study (37). The effect of hyperoxia on the thyroid of adult rats has already been studied by Galton. In that study, male Sprague-Dawley rats showed depressed thyroid activity (i.e., decreased T4 concentration and binding capacity in serum, a decreased rate of deiodination) after the exposition to 40-80% oxygen for 96 h (46). These findings might be explained by the effect of oxidative stress caused by hyperoxia on the thyroid. Oxidative stress results from a disbalance between the formation of radical oxygen species (ROS) and the activity of antioxidative enzymes—a mechanism that also plays a crucial role in the pathogenesis of hyperoxia-induced brain injury (2). The damaging effect of oxidative stress on macromolecules of the thyroid has previously been described (47).

GH is first detectable in the fetal rat pituitary by gestational day 18. The percentage of somatotrophs in the pituitary rises to a peak on P5, when they comprise 40% of all cells in the pituitary (48). Soon after birth there is a GH surge and a decline to adult levels after the first 2 weeks of life (49). Within the 10- to 12-days old rat, GH receptors and binding proteins are widespread in the CNS on neurons and on neural cells such as Purkinje cells, astrocytes and oligodendrocytes, especially in regions involved in neurogenesis such as the hippocampus, the olfactory bulbus and the subventricular zone. Central GH receptors decline by P25 (18). In this study, we found decreased GH levels following hyperoxia, which might be a direct effect of hyperoxia on the somatotropes. The effect of hyperoxia on the somatotropes or other cells of the anterior pituitary during postnatal development remains unclear. To answer the question whether hyperoxia induces injury of the pituitary, especially of the somatotropes, further studies are necessary that investigate markers of oxidative stress (e.g., markers of lipid peroxidation or oxidative injury to nucleic acids) and cell death (50).

Although GH and TSH were only transiently altered in this study, these changes might be clinically relevant as they occur in a vulnerable period of brain development, which is influenced by both the HPT and HPS axes (16, 18, 51). The fact that transient perinatal changes in hormone levels might be relevant for long-term neurodevelopmental outcome is supported by clinical studies which showed an association between neonatal thyroid hormone and GH levels and neurodevelopmental outcome at pre- and school age (25, 36). Further studies are necessary to prove the hypothesis that transient endocrine alterations observed in this study are at linked to the phenotype seen in the model of hyperoxia-induced brain-injury (hypomyelination, microstructural changes, neurocognitive deficits) (7–13).

While changes in GH and TSH were observed in this study, no effects on ACTH as part of the HPA axis were found. This is in contrast to the findings of Kobayashi et al. who found an increased secretion of stress hormones including ACTH upon exposition to hyperoxia (FiO2 100% for 48 h) in 3 months old rats (52). Nevertheless, these rats were much older and considering the age-related response of the HPA axis, the results are not comparable. Studies have described a stress hyporesponsive period until the second week of life in rat pups (53, 54) with blunted ACTH and corticosterone rise. Bruder et al. described an ACTH independent adrenocortical response to hypoxia at P5 (55). Future studies should evaluate the effect of hyperoxia at later time points in HPA axis maturation (beyond P3-P5) and further time points for ACTH measurements should be assessed. In this study, only ACTH (mRNA of POMC and hormone) was assessed as the measurement of corticosterone was considered to be less reliable, as even a short period of maternal deprivation and handling before sacrifice might influence corticosterone concentrations independent of hyperoxia treatment (56).

This study has some limitations. We found transient alterations of the pituitary hormones GH and TSH at P5 in the neonatal rat following hyperoxia. As we focused on pituitary gland hormones, other hormones of the respective axes except of T4 were not studied. Furthermore, we only investigated two time points (P5 and P11). which were chosen on the basis of our previous studies focusing on the acute (P5) and subacute (P11) (oligodendrocyte status equivalent to a term born infant) effects of hyperoxia-induced brain (10, 57). Additional later time points (e.g., in the adolescent and adult rat) are subject to further studies. Keeping the complex phenotype and the multiple origins of pathology of preterm infants in mind, a further limitation results from our single-hit experimental model. Although we found significant alterations in endocrine parameters following the exposition of hyperoxia in a vulnerable period of endocrine development, the clinical significance of these findings has to be proven. Considering the importance of both HPT and HPS axes for neurodevelopment including myelination and cognitive outcome, we hypothesize that transient endocrine alterations contribute to the adverse effects of hyperoxia we repeatedly found in the model of hyperoxia-induced brain injury (7–13).

Nevertheless, the study results are important as they underline the multi-organ effects of high levels of oxygen in the immature organism. They underline the necessity of a rational application of oxygen and further research on optimal saturation limits and monitoring (3). Furthermore, the results underline the fact that research on endocrine changes is necessary as they might open new options for neuroprotection. Both axes may serve as a target for neuroprotective interventions. In a model of hypoxia-ischemia, the application of GH led to reduced neuronal apoptosis in the neonatal rat (58). Also clinical studies hint at neuroprotective effects of GH in children born small for gestational age (59, 60). Although the neuroprotective effect of thyroid hormone was not confirmed in neonatal models (61) or clinical studies (27), a better understanding of thyroid hormone regulation in preterm brain injury is crucial. Although low thyroid hormone levels in preterm infants have been associated with impaired outcome, the causality is still unknown (27). Decreased thyroid hormone levels are also observed in critical ill patients and might be protective in an acute phase of illness (27, 62). A further question is if by the supplementation of T4 sufficient levels of T3 are locally achieved. Thyroid hormone regulation is complex and little is known about the cellular and molecular mechanism thyroid hormone influences brain development—and even less is known about the impact of an interrupted intra-uterine development as it is the case in the preterm infant. Thyroid hormone regulation includes different types of local deiodinases (e.g., in the CNS up to 80% of active T3 is produced locally), thyroid hormone receptors and cell-specific thyroid hormone transporters (16). Further research is necessary to understand thyroid hormone regulation in the preterm infants to adjust, e.g., time point of application with consideration of the clinical state of the infant and the applicated thyroid hormone metabolite in future clinical trials.

## Conclusion

In this study, hyperoxia led to transient endocrine changes in the neonatal rat by altering the activities of the HPT and HPS axes, respectively. Considering the multi-organ effects of hyperoxia on the immature organism, this study underlines the importance of rational use of therapeutic oxygen in neonatal care. Future studies need to elucidate the impact of these transient endocrine changes on neurodevelopment.

## Data Availability Statement

The raw data supporting the conclusions of this article will be made available by the corresponding author upon reasonable request undue reservation.

## Ethics Statement

The animal study was reviewed and approved by the State Agency for Nature, Environment and Consumer Protection North Rhine-Westphalia.

## Author Contributions

MD, HH, and IB: conceptualization and supervision. HH, MK, MD, MS, BM, and ES: methodology. MK: software. IB and UF-M: validation. KM, MS, and MD: formal analysis, investigation, and data curation. IB, HH, and DF-S: resources. MK and MD: writing—original draft preparation, visualization, and project administration. IB, HH, UF-M, BM, and ES: writing—review and editing. MD, IB, and UF-M: funding acquisition. All authors have read and agreed to the published version of the manuscript.

## Funding

This work was supported by the IFORES clinician scientist stipend program of the Medical Faculty of the University Duisburg-Essen, Germany (D/107-40950 to MD); the C.D.-Stiftung and the Karl-Heinz-Frenzen foundation.

## Conflict of Interest

The authors declare that the research was conducted in the absence of any commercial or financial relationships that could be construed as a potential conflict of interest.

## Publisher's Note

All claims expressed in this article are solely those of the authors and do not necessarily represent those of their affiliated organizations, or those of the publisher, the editors and the reviewers. Any product that may be evaluated in this article, or claim that may be made by its manufacturer, is not guaranteed or endorsed by the publisher.

## Abbreviations

ACTH: adrenocorticotropic hormone
ELISA: Enzyme-linked Immunosorbent Assay
GH: growth hormone
HO: hyperoxia
HPA: hypothalamus-pituitary adrenal axis
HPS: hypothalamus-pituitary-somatotropic axis
HPT: hypothalamus-pituitary-thyroid axis
ISH: *in situ* hybridization
NO: normoxia
P: postnatal day
POMC: proopiomelanocortin
TRH: thyroid-releasing hormone
TSH: thyroid-stimulating hormone
T3: triiodothyronine
T4: thyroxine.
